# The applications and techniques of organoids in head and neck cancer therapy

**DOI:** 10.3389/fonc.2023.1191614

**Published:** 2023-06-23

**Authors:** Hao Qi, Xiaolin Tan, Wenshuo Zhang, Yihong Zhou, Shaoyi Chen, Dasong Zha, Siyang Wang, Jinming Wen

**Affiliations:** ^1^ The Cancer Center, The Fifth Affiliated Hospital of Sun Yat-Sen University, Zhuhai, China; ^2^ Department of Urology, The Fifth Affiliated Hospital of Sun Yat-Sen University, Zhuhai, China; ^3^ Department of Clinical Nutrition, The Fifth Affiliated Hospital of Sun Yat-Sen University, Zhuhai, China; ^4^ Department of Oncology, First Affiliated Hospital of Zhengzhou University, Zhengzhou, Henan, China

**Keywords:** head and neck cancer, organoids, techniques, precision medicine, drug testing

## Abstract

Head and neck cancer (HNC) is one of the most common cancers on the planet, with approximately 600,000 new cases diagnosed and 300,000 deaths every year. Research into the biological basis of HNC has advanced slowly over the past decades, which has made it difficult to develop new, more effective treatments. The patient-derived organoids (PDOs) are made from patient tumor cells, resembling the features of their tumors, which are high-fidelity models for studying cancer biology and designing new precision medicine therapies. In recent years, considerable effort has been focused on improving “organoids” technologies and identifying tumor-specific medicine using head and neck samples and a variety of organoids. A review of improved techniques and conclusions reported in publications describing the application of these techniques to HNC organoids is presented here. Additionally, we discuss the potential application of organoids in head and neck cancer research as well as the limitations associated with these models. As a result of the integration of organoid models into future precision medicine research and therapeutic profiling programs, the use of organoids will be extremely significant in the future.

## Introduction

The phrase “head and neck cancer” (HNC) outlines a variety of cancers that affect the larynx, hypopharynx, nasopharynx, mouth, throat, thyroid, and salivary gland. These cancers are categorized based on the anatomical areas in which they develop, including lip, oral, oropharynx, larynx, hypopharynx, and nasopharynx cancers (1). In 2018, HNC was the 7th most prevalent cancer globally (2) with an incidence of more than half a million patients and 300,000 deaths every year (3, 4). Head and neck squamous cell carcinoma (HNSCC), the most prevalent cancer of the upper aerodigestive tract, accounts for over 90% of cases (5) and most of HNC. Other primary sites, such as salivary gland cancers (SGC), encompassing a rare type of HNCs. It is reported that SGC has a wide range of subtypes which preclinical research is lacking. The person-year incidence rate of SGC is less than 3 cases/per 100,000 people (6). In current clinical practice, treatment options include surgery, radiotherapy, chemotherapy, targeted therapy, and immunotherapy. However, HNC therapy is not effective, and various factors cause the high recurrence rates (7). Firstly, ascribing to their anatomic location, hidden lesions complicate surgery. On the other hand, chemotherapy is a crucial part of treatment, but unfortunately, the mechanisms underlying chemoresistance (to drugs like Cisplatin, for example) are still poorly understood (8). Furthermore, relatively high intra-tumor genetic heterogeneity is one of the important factors that lead to highly variable treatment responses. The comprehensive preclinical model which able to evaluate patient responses to therapy plans predictively and identify biomarkers with a high degree of sensitivity and specificity while taking into account interactions between the tumor microenvironment (TME). The preclinical model would make it easier for HNC patients to receive tailored treatment options (9), which link the molecular characteristics of a patient’s tumor with efficient therapeutic measures in a way that is saleable and compatible with systems biology techniques. Over the past few decades, organoid technology has improved the preclinical model and led to an ever-increasing number of breakthroughs toward the promise of precision cancer therapies.

## Organoid and patient-derived organoid

In 2009, Hans Clevers and Toshiro Sato, created the first tiny gut organoids from adult stem cells derived from the gut of mice, setting off a frenzy of organoid research (10). Organoids are microscopic three-dimensional(3D) structures that are grown from stem cells *in vitro*. They recapitulate structural and functional traits of their *in vivo* corresponding organs and possess the capability of self-organize and self-renewal. This technology allows us to simulate complex organ structures and functions *in vitro*, greatly accelerating the development and application of genes and drugs. In the field of cancer research, for now, preclinical cancer research is regularly conducted by immortalized human cancer-derived cell lines (adhesion or suspension) (11). However, it cannot capture tumor architecture, as well as mimic the tumor microenvironment, which is crucial for understanding how cells respond to medications and for designing anti-cancer therapies. Patient-derived tumor xenograft (PDX)is a transplanted tumor model formed by using tumor tissues and primary cells of patients implanted into immune deficient mice. PDX model retains most of the features of primary tumor in histopathology, molecular biology and gene level, and has better clinical efficacy prediction. Therefore, PDX model has been increasingly widely used in many key nodes of new drug development. As PDX preserves the initial intra-tumor heterogeneity and tumor-stroma interactions, they are widely used (12–15). The process of generating a PDX, however, is time-consuming and costly (13).

Therefore, the clinical practice needs a rapid, low-cost, and individual 3D structures tumor model which derived from patients. In 2011, Sato et al. took the lead in establishing colorectal cancer organoids from patient samples with organoid technology (15). This advancing technology is named patient-derived organoid (PDO). PDO has several unique advantages over other model systems. In contrast to static sequencing data, it offers the opportunity to analyze specific tumors as a dynamic system and maintain the characteristics of the original tumor. Due to its biological features, PDO stand in the middle between cell lines and patient-derived xenografts (PDX) models. PDO can more closely resemble a tumor *in vivo* condition while also being simpler to set up and less expensive to maintain. Since the first PDO were created more than ten years ago, many tumor organoids have shown tremendous promise in both clinical and basic research, but head and neck malignancies have seen little use of this technique. Nevertheless, recent studies have revealed that there are several potential uses for head and neck tumor organoids in therapeutic settings.

## Establishment of HNC PDOs

### Construction

The advancement of organoid technology offers new opportunities for pre-clinical field testing of medicative solutions. Technically, the construction steps of most HNC PDOs are similar, with only a few differences, as shown in **Table 1**. For the creation of PDOs, a generic methodology might not be suitable for all types of tumors. Kijima and Karakasheva group described in detail the general establishment process of patient-derived head and neck cancer that most researchers used (18). In brief, after the specimens were washed, minced, and digested, the tissues are dissociated into single-cell suspension. And then mixed with Matrigel for further cultivated. The specific formula of the medium is different (16–18, 20, 22, 25).

**Table 1:** Overview of PDOs in head and neck cancers.

Reference	Disease	Digestive enzyme	Growth scaffold	Methodological improvement
**Noriaki Tanaka (** 16)	HNSCC	Liberase DH	Matrigel	CTOS method
**Else Driehuis (** 17)	HNSCC	Trypsin	Cultrex	NA
**Tatiana A (** 18)	EC/HNSCC	Dispase/Trypsin	Matrigel	NA
**Ren-Bo Ding (** 19)	NC	Collagenase/dispase II/trypsin	Matrigel	NA
**Yoshihiro Aizawa (** 20)	SGC	Liberase/Hyaluronidase	Matrigel	NA
**Gerben Lassche (** 21)	SGC	Collagenase type II/TrypLE Express	Matrigel	NA
**Bo Wang (** 22)	SGC	Dispase	Matrigel	NA
**Xian-Wen Wang (** 23)	NC	Step1: Collagenase II/hyaluronidase Step2: DispaseII/DNase I	Matrigel	Two-step enzymatic strategy
**Zhaohui Wang (** 24)	HNSCC	Not mentioned	Matrigel/Cultrex	Micro-organospheres method
**Hao Yang (** 25)	PTC	DNase I/Collagenase type II	Matrigel	NA
**Kuan-Chou Lin (** 26)	Locally advanced HNC	Not necessary	BCC	CTC method

HNSCC, Head and Neck squamous cell carcinoma; EC, Esophageal Cancer; NC, Nasopharyngeal Carcinoma; SGC, Salivary gland cancer; PTC, Papillary Thyroid Cancer. HNC, Head and Neck Cancer; BCC, Binary Colloidal Crystal; CTC, Circulating Tumor Tells; NA, Not available means that the authors used classical methods of cultivation, without methodological changes.

Based on the work by Kondo et al (27), Noriaki Tanaka et al. constructed the PDOs for HNSCC by using cancer tissue-originated spheroids (CTOS) method in 2018 (16). Unlike traditional strategy, this method requires cultivation in advance in ultra-low culture dishes for 24-72h to form CTOSs. Following verification, CTOSs were moved into Matrigel to form solidoid and cultivated in a medium with added growth factors. The growth rate from original CTOS cultures to organoids is 37.2%. They claimed that the PDO exhibit histological features closely matched those of the donor tumor tissues and that the carcinoma stem cell marker (CD44) expression was similar to those of the original tumors. In order to optimize the digestive conditions, Xian-Wen Wang proposed a new two-step enzymatic strategy for nasopharyngeal carcinoma (23). And they claimed that the success rate is different between primary tumors and recurrence tumors. Zhaohui Wang has developed micro-organospheres technique to provide a more reliable platform for assessing drug response, based on the clinical need for rapid and batch establishment of a large number of organoids using smaller tissue blocks (24). In addition, the Clevers and Driehuis group also created organoids of normal oral mucosa and investigated how the herpes simplex virus infected these organoids (17).

Gerben Lassche et al. presented the first successful development and characterization of SGC PDO cultures. According to their findings, the 19% success rate is rather low when compared to PDOs culture attempts for other cancer types (21). Yoshihiro Aizawa not only established the patient-derived organoid and PDX model of SGC but also built a PDX-derived organoid (PDXO) by using specimens from SGC PDXs. Three subtypes of SGC were successfully generated, and they found these three models showed similar histological features as the original tumors (20). However, salivary gland tumor organoids should be used cautiously in clinical drug sensitivity screening. A case report of a patient with ETV6-NTRK3 gene fusion-positive secretory salivary gland carcinoma which was sensitive to selective TRK inhibitor larotrectinib showed that the drug response in PDOs differs from patient (28). The authors highlight that the growth factors in the medium may cause cellular defense mechanisms to develop that are independent of TRK signaling, which might potentially account for the absence of drug sensitivity.

In addition to using conventional surgical specimens or pathological biopsy specimens to construct PDOs, attempting to construct tumor organoids using circulating tumor cells (CTC) present in liquid biopsies in HNC patients reduces the risks associated with surgery for patients. Kuan-Chou Lin et al. developed the eSelect system to expand CTC ex vivo. Tumor organoids constructed in this way can mimic a patient’s response to clinical drug therapy (26). The success rate of organoid construction is 92.50%, which is much higher than traditional construction methods. This strategy could work for individuals with advanced HNC since many of them had metastatic or incurable conditions that precluded additional biopsies or operations. However, if there are few CTCs present in the early stages of the illness, it could not be effective.

The construction of PDOs has always been the biggest obstacle to its application. Different methods can result in inconsistencies between the produced organoids and the primary tissue. At present, the variation of non-standardized schemes mainly comes from: 1. Different tissue sources (representing only a subset of tumors); 2. Different mediums, growth factors, and poorly defined culture components will unpredictably change organoid phenotypes, leading to differences in growth and biological behavior; 3. The Matrigel varies from batch to batch and contamination will lead to different results. In addition, there are still great differences between *in vitro* culture matrix and actual tumor micro-environment.

### Evaluation of PDOs in HNC

The main difference between tumor and normal tissue is due to genetic heterogeneity. As early as 1953, researchers identified the multifocal origin of squamous epithelial tumors (29), which is the main pathological pattern in HNC. Thus, one of the criteria for evaluating PDOs is whether the genetic characteristics of genes are preserved. The map of genetic mutations in head and neck tumors is extremely complex. Numerous studies have documented frequent chromosomal instability and somatic genomic changes based on data from sequencing technology-based genetic profiling of HNC tissues (30, 31). The inactivation of the tumor suppressor genes TP53, CDKN2A, and PTEN, as well as the amplification of the CCND1 gene, are the primary genetic modifications that turn dysplasia into invasive HNSCC (32). In addition, following bulk sequencing investigations, it was discovered that HNSCC frequently has mutations in TP53, FAT1, CDKN2A, PI3KA, and NOTCH (31, 33). Also, genetic alteration profiles may differently depend on HPV status. While CDKN2A and TP53 mutations are the most frequent in HPV-negative cancers, HPV-positive tumors frequently include TP63, TRAF3, and E2F1 mutations (31, 33, 34). Based on multiple types of mutations, De Cecco et al. proposed six distinct subtypes (immunoreactive, inflammatory, HPV-like, classical, hypoxia-associated, and mesenchymal) in HNSCCs (35). There is also a strong link between genetic mutation and treatment response. More recently, study showed HRAS mutations in HNC make tumors more vulnerable to farnesyltransferase inhibitor tipifarnib (36). Another high-frequency mutation of PI3K leads to a wide range of applications of PI3K inhibitors in HNC (37–39). Heterogeneity is difficult to reproduce in 2D culture, and the PDX model may also have gene changes due to the doping of the mouse tumor microenvironment. Due to their ability to mimic the genetic changes associated with a primary tumor, organoids have many advantages.

In the case of HNC, an ideal marker is required to describe molecular changes in tumor cells and predict therapeutic targets. PDOs provide a way to describe the genetic map of tumors at different stages of the different expression patterns of tumor heterogeneity. Almost all studies on HNC PDOs have used immunological methods to assess the genetic similarity to primary tumors (16–18, 27, 40), and unanimously described that organoids retain intra-tumoral heterogeneity of the original tumor. As shown in **Table 2**, most studies used immunostaining to detect the expression of some markers, for example, TP53, Ki67, KRT5, and CD44 (16–18). Whole-exome sequencing (WES) detected common mutations in HNC PDOs, such as TP53, PIK3CA, BRAF, and CDKN2A (17). Single-nucleotide variants (SNV) and small insertions or deletions (Indel) throughout the genome were compared between organoids and primary tumors (19). And simultaneously it was found that organoids retained the chromosome missegregation of the primary tumor well. Genetic uniformity of salivary gland carcinoma PDOs also have been evaluated by sequencing (20). Therefore, PDOs largely recapitulated the genetic alterations that were detected in the tumor. In addition, it was shown that PDOs retained their tumorigenic potential upon xenotransplantation (20).

**Table 2:** An overview of application in HNC PDOs.

Reference	Tissue source	Patient/cases	Success rate	Marker	Therapy evaluation			Annotation
					Chemotherapy	Targeted therapy	Radiotherapy	
**Noriaki Tanaka** (16)	HNSCC	43	16/43(37.2%)	Pankeratin; CD44; ALDH1A1	Cisplatin; Docetaxel	–	–	–
**Else Driehuis (** 17)	HNSCC (oral cavity, pharynx, larynx, salivary gland, nasal cavity, and neck)	31	31/31(60%)	TP40; TP53; MKI67; KRT5	Ciaplatin; Carboplatin	Cetuximab	+	Other treatment: Chemo+RT; Everolimus; AZD4547; Niraparib
**Else Driehuis (** 41)	HNSCC (Tongue; larynx; parotid gland; oral cavity; gingiva)	8	7/8(87.5%)	EGFR	–	Cetuximab; 7D12; 7D12-9G8	–	–
**Tatiana A. Karakasheva (** 18)	Esophageal Squamous Cell Carcinoma (ESCC)	1	-	Ki67; p53; SOX2; CDX2	Cisplatin; Paclitaxel	-	-	-
**Ren-Bo Ding (** 19)	Nasopharyngeal Carcinoma (Epithelial; Sarcomatoid; Mixed)	43	40/43(93%)	AE1/3; Vimentin/LMP1	Docetaxel; Paclitaxel;	Gefitinib	+	Drug library screen
**Sasidharan Swarnalatha Lucky (** 42)	Nasopharyngeal Cancers	18^#^	-	EBV; CD44	-	-	+	Radiation Dose Optimization
**H. Zhao (** 43)	Oral Squamous Cells Carcinoma	12	12/12(100%)	CD44; CD133; SOX2	–	–	–	CAF model
**Yoshihiro Aizawa** (20)	Salivary Gland Carcinoma	35	4/35(11.4%)	CK; AR; HER2; GCDFP-15	-	-	-	-
**Gerben Lassche (** 21)	Salivary Gland Carcinoma	37	7/37(19%)	CK7; P63; AR; HER2	Cisplatin	Lapatinib; Erlotinib; Sunitinib	–	–
**Kuan-chou Lin (** 26)	Head and Neck Cancer	40	37/40(92.5%)	EpCAM; CD45	Cisplatin; 5-FU; Docetaxel	-	-	-
**Xian-Wen Wang (** 23)	Nasopharyngeal Carcinoma	62	39/62(62.9%)	CD133; CD44; BMI-1; EBERs	–	–	–	–

The symbol in the table indicates that it has been applied to organoids (+) and has not been applied to organoids (-). Chemo, Chemotherapy; RT, Radiotherapy; CAF, Cancer-Associated Fibroblast; ^#^PDX-derived organoid

Next, the drug response of PDOs is also an important aspect of evaluation, which is also the premise of whether these models can be personalized and applied to patients’ treatment prediction. More than 60% of patients diagnosed with head and neck cancer tumors are in the local advanced stage and therefore require multi-mode treatment such as surgery combined with radiotherapy and chemotherapy (44). Even so, 65% of patients had a relapse (45), which need more effective therapy. In recent years, the rise of targeted therapy and immunotherapy offers hope for patients. Cetuximab has been established as first-line standard-of-care therapy for recurrent or metastatic head and neck cancer (46). Additionally, the approval of programmed death 1(PD-1) immune-checkpoint inhibitors makes the treatment of HNC is more promising. Unfortunately, the objective response rate remains low (46–48). Moreover, the treatment of patients with advanced head and neck tumors is highly heterogeneous, and the prediction based on protein marker expression is controversial (2). Therefore, *in vitro* models that can simulate patients are needed to test drug sensitivity. Current studies have shown that *in vitro* testing of HNC PDOs reveals that they respond to chemotherapeutics, targeted treatments, and immunological agents (16, 17). Most studies of organoids in head and neck tumors have tested susceptibility to chemotherapy, for example, cisplatin. There have also been individual studies that have tested the sensitivity of targeted EGFR therapy (See **Table 2**). However, due to the limitations of organoid culture, no PDOs studies have been conducted to probe the sensitivity of immunotherapy in HNC. Advances in culture technology allow us to create PDOs that are smaller with a larger surface area (24), which opens up the possibility of immunotherapy tests in the future.

It is worth remembering that conventional colony-forming assays, which are commonly used to assess 2D cell proliferation conditions, were inapplicable for PDOs because the size changed so inapparently after treatment, even though there was significant cell death in particular. There have been some ambiguous results for cell viability tests due to 3D growth and the existence of complex cell clusters, such as Celltiter-Glo (16, 41, 49). Simple and straightforward methods are needed to detect organoid drug responses. ATP-based end-point luminescence assays(Celltiter-Glo) used to consider to be a better option to assess viability of 3D cultures (50). When the organoid whole volume is observed at the single-cell level, A novel method that evaluates cell metabolism utilizing intrinsic fluorescence from NAD(P)H and FAD on a single cell level for a 3-D *in vitro* model was tested in HNC PDOs (51).

## Technological improvement

**Figure 1:**
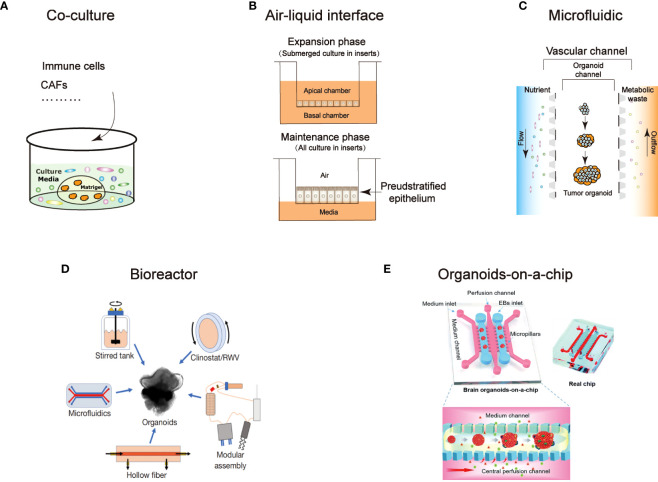
An overview of technological developments in organoids. Characterize the different technical features: **(A)** Co-culture **(B)** Air-liquid interface **(C)** Microfluidic **(D)** Bioreactor (94) **(E)** Organoids-on-a-chip (95).

Organoid culture technology advancements have both sped up mass production culture and improved simulations of the microenvironment in the human body. Here, we summarized the present state of organoid culture technologies (**Figure 1**) and discussed the potential applications for them in PDOs of head and neck tumors (**Table 3**).

**Table 3:** An overview of technologies that have been used for organoid.

Culture Techniques	Definition	Target Problem	Advantage	Disadvantage	Employed in HNC	Reference
**Co-cultural**	Stromal cells, immune cells, or other chemicals are added to the culture medium of the constructed organoids.	Study interaction between organoids with stromal cells or immune cells	1.Simple and convenient to operate. 2. Achieve the preliminary cell-to-cell interaction	1.Additional cells and growth factors are needed. 2.Lack of extracellular matrix components 3.nonhomologous culture results in immunohyperreactivity	Yes	(43, 52)
**Air-liquid interface**	This method seeds cells on a collagen-coated transwell membrane. After apical and basolateral surfaces epithelium is established, the apical medium is removed and cells are fed through a porous membrane from the basolateral surface.	Study interaction between organoids with stromal cells or immune cells	1.Provide an effective oxygen supply to organoids 2.Able to retain the original tissue structure 3.Stromal cells provide nutritional factors without additional supplementation	1.The immune components cannot be sustained for long (<2 months) 2.Determining optimal cultivation conditions is costly in terms of manpower and time 3.There were hypoxia and necrosis in large organoids	No	(52–54)
**Microfluidic device**	A device system manipulates a small amount of fluid at the submillimeter length scale. It provides dynamic culture conditions by supplying continuous inflow and outflow of medium and nutrition for organoids.	A structured culture system was used to simulate vascularization perfusion with a 3D print device	1.Capable of exogenous controlled addition of cells, growth factors and chemical substances. 2.Cellular diversity is preserved in organoids 3.Less culture-medium and growth factors are required 4.Simulated perfusion shear force	1.The material fabrication process affects repeatability and effectiveness. 2.Microfluidic device materials affect immune response 3.Determining the growth factors and/or inhibitors required to maintain all subclones is the laborious and time-consuming process;	Yes	(55–57)
**Bioreactor**	A dynamic 3D cell culture platform that rapidly supplies cells with nutrients and growth factors that promote cell proliferation and differentiation	Mechanical devices were used to enhance the growth rate of organoids, and organoid models were constructed rapidly and in large numbers	1.Obtain organoids quickly and in large quantities in a short time 2.Good repeatability	There is a wide variety of equipment, there is no unified standard, different tissue organoids need different equipment	No	(58–61)
**Organoids-on-a-chip**	An integration of organoids with organ-on-a-chip technology. Organ-on-a-chip is a microfabricated cell culture devices designed to model the functional units of human organs *in vitro*. This combination makes good use of precise microenvironment control in organ-on-a-chip model.	The accurate regulation of the microenvironment in organoid development.	1.Microenvironmental control of organoids 2.Tissue-tissue and multiorgan interactions 3.Reducing variability	Limited ability to recapitulate dynamic structural, environmental, and functional changes that occur during organogenesis	Yes	(62–64)

### Co-culture

Studies on cancer therapy are limited as a result of the inability of *in vitro* models to analyze the connection between the vasculature and the stroma (52). Co-culture method could retain natural stromal elements, such as different immune cells, or by incorporating foreign immune cells, cancer-associated fibroblasts (CAFs), vasculature, and other elements, which could closely mirror the tumor microenvironment (65). The expression of traditional mesenchymal markers and EMT-associated genes in partial HNSCC cells supports the idea that cancer cells and CAFs interact with one another in a regulatory manner (66). On this point, Xu Chen reported the method for the model of fibroblast-attached organoid to investigate the contact-dependent mechanisms (67). The mix cluster of single fibroblasts and oral squamous cell carcinoma cells were resuspended in medium in an ultra-low attached (ULA) plate before co-culture in Matrigel. In this model, they revealed that the stimulation of fibroblasts associated to the oral squamous cell carcinoma PDOs is mediated by Notch pathway. Coincidentally, another study reported that co-culture of CAFs with oral squamous cell carcinoma PDOs promotes stem-like properties of tumor (43). Hui Zhao et al. used co-culture method to demonstrated nicotinamide N-methyltransferase in cancer-associated fibroblasts is corelated with tumor growth and extracellular matrix remodeling is a potential therapeutic target for patients with oral squamous cell carcinoma (65). Moreover, co-culturing PDOs with immune cells or PBMCs can simulate features of the tumor immune regulation, such as T cell activation, infiltration into tumors, T cells recognition, and cancer cell eradication (52). Consequently, co-culture technique is a good platform for CAR-T (chimeric antigen receptor T-cell immunotherapy) evaluation (68, 69). Tsai et al. created pancreatic cancer PDOs using co-culture method with patient-matched peripheral blood lymphocytes and CAFs to study relevant between the immunotherapy and tumor-immune cell interaction (70). In colon cancer, Theresa E et al. provided the evidence that CAR-engineered NK-92 cells exhibit tumor antigen-specific cytotoxicity in organoid (71). However, the cytotoxic effects of T cells in PDOs have not been studied in the field of head and neck cancer, adoptive cell therapy advancement recently may provide opportunity for new perspective (72). It will be appealing to employ co-culture systems in the future to anticipate the efficiency of immunotherapy since several studies have shown that it is beneficial for HNCs (73–75).

### Air-liquid interface

To explore patterns in how tumors interact with the microenvironment, the ALI technique is designed for the evaluation of the interaction between the epithelium and stromal microenvironment. Two dished (inner and outer) were used to separate collagen gel matrix and culture media in which the growth of organoid tissue retain native tissue architecture without reconstitution (53). And the organoid constructed in this way does not need to add additional growth factors because the endogenous factors secreted by stromal cells are sufficient (76). ALI technique produces organoids that include immune cells like T cells and B cells adjacent to the tumor epithelium (52) and they successfully mimic immune checkpoint inhibition by inhibiting PD-1/PD-L1, activating tumor antigen-specific tumor-infiltrating cells, and generating tumor cytotoxicity (52). It has so far been applied to a number of cancers, such as kidney (77) and colorectal cancer (77, 78). Despite the fact that the anticancer effect of tumor-infiltrating lymphocytes offers immune treatment strategies for the prevention of HNCs progression to improve patients’ overall survival (79, 80), ALI technology has not been tested in HNC PDOs. As the effectiveness of immune medicines in HNC PDOs has not yet been established, the ALI technology offers a promising avenue of investigation.

### Microfluidic

Photolithography and 3D printing technology allows us to accurately build frameworks that simulate microenvironments in organism (58). The scientific progress of devices that control a submillimeter volume of fluid is known as microfluidics. Organoids and cultural medium are distributed in different channels, continuously feeding the organoids through inflow and outflow by microfluidic device (81). It can be used to investigate stromal cell cross-talk and T cell penetration into cancer organoid by adding exogenous T cells into the media channels (82). Recently, custom designed 3D printed microfluidic chip co-culture technique provide possibility to *de novo* generated vasculature with cerebral organoids (55, 83). It is a simple, extremely efficient method of vascularizing organoids that may be used with any organoid system. This microfluidic technique has been used to successfully culture up to two types of organoids(liver and islet) and detect metabolism-related pathway activity (84). Well-designed microfluidic devices that maintain autologous myeloid and lymphoid cell populations similar to the original donor can be used to detect dynamic response and resistance to immune checkpoint inhibitors (85). However, the materials used in the microfluidic devices are different from those used in microscopic cultures, making generalizations difficult (56). In fact, cells cultivated on polystyrene or glass still form the basis of the bulk of newly published research on *in vitro* cell biology. Zhaohui Wang recently designed a microfluidic device to generate nanoliter-sized droplets contains organospheres, and in this way they were able to rapidly and massively produce organoids for head and neck tumors (24).

### Bioreactor

Bioreactor is a device that allows cells or tissues to grow in a controlled environment. Through a series of mechanical regulations, the cells are provided with sufficient nutrients and a suitable environment in which cells or tissues can grow rapidly. Specific and detailed techniques can be referred to Xuyu Qian et al.’s work (59). In many studies, bioreactors have been shown to significantly enhance the efficiency of 3D culture (86, 87). The rapid cultivation of large numbers of organoids in small samples is a widely faced problem. The HNC biopsy specimens are frequently too small and inadequate to establish direct organoids. Additionally, restrictions in the transport of oxygen and nutrients may impede the formation of organoids in static cultures. It has been reported that rotating-wall vessel bioreactors (88) and bioreactor SpinΩ (58) can rapidly promote retinal organoid and brain organoid production respectively. The development of materials technology has provided a great impetus for the translation of basic research into the clinic, new-type bioreactor may be possible to solve the problem in expanding the culture of organoids from small biopsy tissue.

### Organoids-on-a-chip

Organoids-on-a-chip is an integration which combines the advantages of organoids and organ-on-a-chip technology. This is a microfabricated cell culture devices designed to imitate the functional units of human organs *in vitro (* 89, 90). This model can mimic integrated organ-level functions necessary for physiological homeostasis, as well as complex disease processes (90, 91) by integrating living human cells with synthetically generated yet physiologically relevant micro-environments, and furthermore, simulate multiorgan interactions and physiological responses at the systemic level (62). The precise micro-environment control in this technology opens up a new idea for organoid and provides solutions to several problems. The precise control of micro-environment is the main barrier in organoid development, in which contains biochemical signaling, mechanical forces and nutrient supply. Yuli Wang et al., utilized a cross-linked collagen hydrogel to form a biomimetic scaffold, in which small intestinal epithelium cells were guided to form a crypt-villus architecture. Cells polarized by application of gradients of soluble biochemical reagents along the crypt-villus axis (92). Homan et al. used a 3D printed chamber to form a kidney organoid-on-chip model. He and his co-worker found out that high fluid flow stress (FFS) resulted in acceleration of hPSCs-derived kidney organoid vascularization and maturation of glomerular and tubular compartments. Additionally, concurrent morphogenesis of podocytes and tubular epithelial cells was accelerated in a shear stress-dependent manner, indicating that the flow-generated microenvironment was a key factor in the structural and functional development of the kidney organoid (93). Despite micro-environmental control, organoids-on-a-chip technology also achieve modeling biological interactions and reducing variability (63). Although there is no definitive organoids-on-a-chip study in head and neck tumor research, but Zhaohui Wang et al. have developed a chip to produces head and neck tumor organoids rapidly (24).

## Application

### Prediction of treatment effectiveness

40% of patients with HNSCC relapse despite conventional therapeutic approaches, in part because they lack the effective strategy to choose the sensitive treatment. To forecast patients’ response to therapy *in vitro* is one of the most crucial uses of PDOs. The outcomes offer specific directions for clinical patient care. In HNC PDOs, several studies have explored the sensitivity of cisplatin (16–18) and found that this sensitivity could simulate patient response to treatment partially. One HNC subtype, nasopharyngeal cancer, maintains unique molecular characteristics, medication response, and graded radiation sensitivity (19). Potential subtype-specific therapy regimens are identified through the use of PDO-based pharmacological tests. It is reported that the epithelial subtype is more sensitive to EGFR inhibitor and sarcomatoid subtype and mixed sarcomatoid-epithelial is more sensitive to microtubule inhibitors (19). In addition to chemotherapeutic drugs, targeted drugs is also used to test organoids for drug sensitivity. Epidermal Growth Factor Receptor (EGFR) protein level is much higher in head and neck tumors than in normal tissues, making it an effective therapeutic target. EGFR-targeted photodynamic therapy (PDT) could successfully kill head and neck tumor organoids and does not affect organoids of normal tissue formation due to low EGFR expression level in the latter (41). Radiotherapy is an effective treatment for head and neck tumors, so it is of great significance to predict the sensitivity of radiotherapy for clinical treatment. Researchers Driehuis and colleagues found a connection between the clinical outcome of the relevant donor patient and the HNSCC tumoroid’s radiation sensitivity. In patients who relapsed after radiotherapy, the corresponding organoids showed the same resistance to radiotherapy (17). Furthermore, the HNC PDOs can also be used to establish radiobiological parameters (42). On this detail, A technique for medium-throughput drug screening using HNSCC and colorectal PDOs was reported by Putker and his colleagues in 2021. They stated that any tissue-derived organoid model with *in vitro* exposure to chemotherapy and/or radiation can use this platform (96).

However, the use of PDOs to predict therapeutic outcomes is still in its infancy. These results need to be interpreted with caution, although many researchers have produced organoid drug responses similar to those in patients. Appropriate gene-targeting mutations do not necessarily mean that specific drug therapy is effective. For instance, on one occasion an organoid responded favorably to a PI3K inhibitor despite the absence of a PI3K gene-activating mutation in the organoid (17). As a result, functional research may occasionally be more insightful than genetic research.

### Biobank construction and biomarker prediction

The Cancer Genome Atlas (TCGA) and the International Cancer Genome Consortium used a large number of tumor gene expression profiles to create a comprehensive “atlas” that provides a powerful tool for understanding tumorigenesis, progression, and therapeutic response (97, 98). Theoretically, PDOs provide a faster and more convenient way to obtain tumor expression information and to obtain expression profiles at any stage and location, especially in patients with advanced tumors. Patients with HNCs are characterized by numerous subtypes and obvious heterogeneity. In some small sample studies, PDOs recapitulate genetic alterations found in HNSCC patients (16, 17, 19, 28). For instance, PIK3CA mutations and the lack of EGFR amplifications, two characteristics of HPV-positive HNSCC that are lost in established cell lines, are retained in PDOs (99). In addition, biobank established using PDOs can also be used as a biomarker prediction for sophisticated tumor subtypes. Bo Wang et al. established a patient-derived organoid biobank with salivary gland tumors (benign and malignant) to explore the subtypes of tumors. The biobank replicated the transcriptional and anatomical characteristics of the original malignancies, which uncover PTP4A1 as a mucoepidermoid carcinoma diagnostic biomarker (22). As a result of the use of PDOs in the future, it is expected that more “atlases” will be established for the guidance of clinical treatment.

### Studying the risk factor of HNC using PDOs

Research on humans and animals has been hampered by questions of access to samples and ethics. Fortunately, organoids are a powerful alternative, preserving genetic and phenotypic information, and simulating the progression of tumors and metastasis (100). The ability to be expanded *in vitro* rapidly distinguishes tumoroids from PDX models and 2D cell cultures, allowing for the quick production of a large number of clones for further research on clinically orphan disease (101). And organoid system serves as a physiologically relevant experimental platform to determine the effects of epithelial exposure to harmful environmental chemicals such as alcohol (102) and acetaldehyde (103). Shimonosono et al. studied the mechanism of ethanol exposure by HNC organoids in 2021 since alcohol use is one of the major risk factors for HNC (104). The results demonstrated in non-CD44H cells, mitochondrial damage is induced by oxidative stress and apoptosis. Meanwhile, ethanol exposure improved intratumorally CD44H cells growth in an autophagy-dependent way. At present, there are very few basic studies applied to head and neck tumor organoids. It is expected that more studies will follow with the progress of modeling methods.

## Limitation and perspective

### Limitation

In recent years, tumor organoids have developed rapidly, but deficiencies and challenges still exist. First and foremost, the success rate of PDOs construction varies across tumor pathological types, sometimes, rather low (105). Underlying factors contribute to the low success rate, such as non-standard culture medium, pathological type of tumor, cancer heterogeneity, and lack of standardized protocols. Therefore, a standard tumor organoid procedure for HNC is required, in which the sample processing, digestion, and culture conditions of different tumor types should be specified. Of course, this will consume a lot of time cost and labor cost to build. Notably, a study has shown that optimization of the culture medium composition could partially improve organoid growth in breast cancer (106).

Secondly, whether the PDOs are truly representative of the primary tumor and whether they introduce unexpected bias while cultured *in vitro* remains unknown. Under some circumstances, the dominant growth of epithelial cells contamination could be detrimental for cancer cells in the organoid cultures and is a problem in some cancers (107). ALK inhibitor A83-01 (108) and p38 inhibitor SB202190 (109) are two typical additives to organoid culture media that may interact with medications that target the same signaling pathway. Consequently, like the CCK-8 assay in 2D cell culture, the development and validation of a universal and reliable protocol with predetermined threshold values for medication response will be necessary for the therapeutic application of PDOs. In addition to technical obstacles, PDOs do not comprise the tumor microenvironment which is consist of fibroblasts or immune cells. The emergence of co-cultures, which are detailed in more detail above, has partially addressed this.

Last but not least, tumoroid lack of vasculatures and neuronal networks restrains their capability to be used as precise models to investigate the effects of personalized therapeutic strategies. Technically, some of the technologies mentioned above are attempting to address this shortcoming, such as co-culture method and air-liquid interface, but there is still much work to be done. Furthermore, before adoption in clinical cancer treatment can be taken into consideration, there are still numerous challenges to be solved, and large cohort investigations are urgently required.

### Perspective

In the past, it took at least 10 years for a drug to be developed into a clinical application. The use of organoids has greatly accelerated the transfer of drugs from the bench to the bedside. An ongoing multicenter observational study (NCT04261192) is going to assess the feasibility of using PDO from HNSCC as tools for predicting response to treatments. The study plans to verify the sensitivity and clinical predictive value of organoids to chemotherapy, radiotherapy, RAPR inhibitors, and immunotherapy. And new targets are predicted based on molecular signatures and therapeutic response (110). Of note, this study will coculture of PDO with immune cells. Another clinical trial (NCT05400239), which has not yet begun recruiting, is planned to assess whether patient derived organoids can be used to predict treatment sensitivity in HNC patients. The completion of clinical trials will promote the further application of head and neck tumor organoids in precision therapy. Narmal tissue organoids can be used to better understand and estimate treatment-related adverse effects, which are frequently seen with targeted therapy. This use is less well known. For instance, E Driehuis et al. studied the effect of MTX on oral mucosa organoid (111). Another noteworthy example is the investigation of nephrotoxic medicines using kidney organoids (112).

Recently, the application of genetic engineering can enable us to better understand the mechanism of tumor occurrence and progression at the genetic level. As a powerful genome engineering tool, CRISPR/Cas9 may be used to modulate genes in organoids to deliver various target gene mutations for further underlying mechanism research (105). In other words, this technique can introduce oncogenic mutations for simulating tumorigenesis. Due to the urgent need for a thorough understanding of the various mutational drivers of the specific cancer subtype, which may not be available, there are significant challenges in the implementation of this technique. There have already been reports of successful use of CRISPR/Cas9 in tumor organoids, such as colorectal, breast, glioma, and ovarian (113–115). It might represent a promising approach to modeling oral carcinogenesis. Additionally, organoid technology can be used to evaluate people who have a family history of head and neck cancer to rule out major risk factors in their lifestyle, such as smoking, drinking, and being susceptible to HPV infection, thereby reducing the likelihood of potentially disastrous events like invasion. Regeneration of surgical tissues by organoids can give HNSCC patients with autologous transplantation and improve the quality of their lives in addition to early detection. PDOs may provide a platform to facilitate investigation into the utility of novel therapeutic strategies, including CAR-T cell therapies (71) and oncolytic virotherapy,

As PDOs research expands significantly, there is an increased potential for understanding aspects of cancer, stratification of patients, and the development of personalized therapeutic strategies based on these findings. However, to fully exploit its potential, it is necessary to further adapt the state-of-the-art methods and technologies related to organoids. It is imperative to continue the quest for biologically relevant inquiries and technical progress, designing studies crucial to patients. The expansion of collaborations and bio-bank sharing is also critical to increase the sample size of our cohorts and apply the appropriate expertise. To conclude, the validation of organoid-derived data may help develop precision medicine approaches that can optimize health outcomes in patients with head and neck cancer by providing meaningful results that can potentially be used in diagnostics and therapeutics.

## Author contributions

HQ, XT, WZ, YZ, and DZ wrote the manuscript. SC and SW contributed to the limitation and perspective. JW revised the manuscript and responsible for the overall content. All authors contributed to the article and approved the submitted version.
